# Protective Immunity Induced with the RTS,S/AS Vaccine Is Associated with IL-2 and TNF-α Producing Effector and Central Memory CD4^+^ T Cells

**DOI:** 10.1371/journal.pone.0020775

**Published:** 2011-07-11

**Authors:** Joanne M. Lumsden, Robert J. Schwenk, Lisa E. Rein, Philippe Moris, Michel Janssens, Opokua Ofori-Anyinam, Joe Cohen, Kent E. Kester, D. Gray Heppner, Urszula Krzych

**Affiliations:** 1 Division of Malaria Vaccine Development, Walter Reed Army Institute of Research, Silver Spring, Maryland, United States of America; 2 GlaxoSmithKline Biologicals, Rixensart, Belgium; New York University, United States of America

## Abstract

A phase 2a RTS,S/AS malaria vaccine trial, conducted previously at the Walter Reed Army Institute of Research, conferred sterile immunity against a primary challenge with infectious sporozoites in 40% of the 80 subjects enrolled in the study. The frequency of *Plasmodium falciparum* circumsporozoite protein (CSP)-specific CD4^+^ T cells was significantly higher in protected subjects as compared to non-protected subjects. Intrigued by these unique vaccine-related correlates of protection, in the present study we asked whether RTS,S also induced effector/effector memory (T_E/EM_) and/or central memory (T_CM_) CD4^+^ T cells and whether one or both of these sub-populations is the primary source of cytokine production. We showed for the first time that PBMC from malaria-non-exposed RTS,S-immunized subjects contain both T_E/EM_ and T_CM_ cells that generate strong IL-2 responses following re-stimulation *in vitro* with CSP peptides. Moreover, both the frequencies and the total numbers of IL-2-producing CD4^+^ T_E/EM_ cells and of CD4^+^ T_CM_ cells from protected subjects were significantly higher than those from non-protected subjects. We also demonstrated for the first time that there is a strong association between the frequency of CSP peptide-reactive CD4^+^ T cells producing IL-2 and the titers of CSP-specific antibodies in the same individual, suggesting that IL-2 may be acting as a growth factor for follicular Th cells and/or B cells. The frequencies of CSP peptide-reactive, TNF-α-producing CD4^+^ T_E/EM_ cells and of CD4^+^ T_E/EM_ cells secreting both IL-2 and TNF-α were also shown to be higher in protected vs. non-protected individuals. We have, therefore, demonstrated that in addition to TNF-α, IL-2 is also a significant contributing factor to RTS,S/AS vaccine induced immunity and that both T_E/EM_ and T_CM_ cells are major producers of IL-2.

## Introduction

Immunologically based resistance against many pathogens is often multi-factorial and involves both cellular and Ab mediated mechanisms. Protracted protection, therefore, requires the generation and maintenance of Ag-specific memory T and B cell responses. In this regard, CD4^+^ T cells may be considered as central mediators of protective immunity because they not only help to promote CD8^+^ T cell and B cell immune responses, but they also provide their own necessary effector function [1], [2]. Although there have been many studies in humans on the capacity of various vaccines to induce and maintain memory T cell responses against viral and other diseases [3], [4], [5], there has been only limited analyses of memory T cell responses induced by vaccines against protozoan pathogens such as *Plasmodium falciparum* (Pf) malaria, a disease that causes over one million deaths annually. It is clear, however, that memory T cell responses to pre-erythrocytic stages are not readily detectable in subjects naturally exposed to malaria infection [6]. One of the goals of a successful anti-malaria vaccine, therefore, is to circumvent this problem and to promote the induction of long-lived memory T cell responses against re-infection.

RTS,S, which consists of the repeat (R) and C-terminal region of the Pf major circumsporozoite protein (CSP) fused to the surface (S) Ag of Hepatitis B virus and co-expressed with free S Ag [7], is currently the only sub-unit vaccine that provides protection against malaria. The RTS,S/AS vaccine confers sterile protection in approximately 40% of malaria naïve subjects exposed to experimental challenge with Pf sporozoites [8]. In clinical trials in infants and children living in malaria endemic regions in sub-Saharan Africa, RTS,S/AS was shown to protect 30% [9] to 66% [10] of young children against first or only episode of clinical malaria, with clinical efficacy extending up to 43 months following vaccination [11]. The vaccine is currently being tested in a phase III trial involving 11 sites in 7 countries in East and West Africa.

A phase 2a RTS,S/AS vaccine trial [8] was conducted at the Walter Reed Army Institute of Research involving 80 subjects. Sterile immunity against a primary challenge with infectious sporozoites was observed in 40% of individuals. Analyses of Pf CSP-specific cellular responses demonstrated that on the day of challenge the frequency of CSP peptide-reactive CD4^+^ T cells producing any combination of 2 or more of the following bio-markers: IL-2, TNF-α or IFN-γ and CD40L, was significantly higher in protected subjects as compared to non-protected subjects. However, we did not address whether the RTS,S/AS vaccine induced memory CD4^+^ T cells and if these memory cells might be associated with protection. The RTS,S/AS vaccine trial affords a particularly unique opportunity for studying memory T cells: a large cohort of immunized protected and non-protected subjects; samples of peripheral blood mononuclear cells (PBMC) from multiple time points of vaccination, including baseline as well as samples following immunizations and challenge.

It is now well established that Ag-experienced CD4^+^ T cells form discrete sub-populations of memory cells that include long-lived, self-renewing CD45RO^+^CCR7^+^ T central memory (T_CM_) cells and relatively short-lived CD45RO^+^CCR7^−^ T effector/effector memory (T_E/EM_) cells [5]. For viral infections, it has been demonstrated that T_CM_ cells produce predominately IL-2 while T_E/EM_ cells secrete primarily IFN- γ [12], [13], [14]. The relative amounts of cytokines produced by these two populations of cells, however, may depend on the nature of the vaccine, the strength and persistence of the antigenic signal, as well as other factors.

In the present study, we asked whether the RTS,S vaccine induces CSP-specific CD4^+^ T_E/EM_ and/or CD4^+^ T_CM_ and if these populations produced similar or distinct cytokine responses and finally whether the cytokine profile of one or both of these subpopulations could serve as an immune correlate of protection against experimental challenge with Pf sporozoites. Our results demonstrated for the first time that PBMC from RTS,S-immunized subjects contain both T_E/EM_ and T_CM_ cells that generate strong IL-2 responses following re-stimulation *in vitro* with CSP peptides. Moreover, both the frequencies and the total numbers of IL-2-producing CD4^+^ T_E/EM_ cells and of IL-2-producing CD4^+^ T_CM_ cells from protected subjects were significantly higher than those from non-protected subjects. We also demonstrated for the first time that there is a strong association between the frequency of IL-2-producing CD4^+^ T cells and the titers of CSP-specific antibodies in the same individual, suggesting that IL-2 may contribute to protection by promoting both cellular and humoral immune responses.

## Materials and Methods

### Study protocol

The study protocol for the phase 2a double-blind, randomized, challenge trial of the RTS,S/AS vaccine conducted at the Walter Reed Army Institute of Research has been described previously [8]. Briefly, 80 healthy malaria-naïve adults received RTS,S/AS at 0, 1 and 2 mo, and then underwent challenge with *P. falciparum* infected mosquitoes. PBMC were collected at pre-immunization (Pre), 2 wk post 2^nd^ immunization (Po2), 2 wk post 3^rd^ immunization (Po3) = day of challenge (DOC), and 2 mo post challenge (PoCh).

### Ethics Statement

A written informed consent was provided by study participants and/or their legal guardians. The study protocol was approved by the WRAIR Human Use Review Committee and the US Army Medical Research and Materiel Command's Human Subjects Research and Review Board (Fort Detrick, Maryland).

### PBMC collection

PBMC were isolated as described previously [8] and maintained in liquid nitrogen until use.

### Antigens

Pf CSP peptides (Alpha Diagnostics, San Antonio, TX) corresponding to the C-terminal of the CSP region of RTS,S consisted of two contiguous peptides designated P2, the 44 a.a. peptide EEPSDKHIKEYLNKIQNSLSTEWSPCSVTCGNGIQVRIKPGSAN and P4, the 35 a.a. peptide KPKDELDYANDIEKKICKMEKCSSVFNVVNSSIGL.

### Intracellular cytokine staining

PBMC from all time points for a single individual were analyzed in a single assay. Typically, PBMC from one protected and one non-protected subject were examined per assay. Briefly, PBMC were washed twice and re-suspended in culture medium consisting of RPMI 1640 supplemented with 100 IU/ml penicillin, 100 µg/ml streptomycin, 8 mM glutamax, 1 mM nonessential a.a., 1 mM sodium pyruvate, and 50 µM 2-ME and containing 20% human AB serum (Gemini Bioproducts; Woodland CA). The cells were then stimulated for 2 h in 96 well U-bottom bottom plates with 2 long contiguous CSP peptides (at a final concentration 20 µg/ml of each peptide per well) in the presence of 1 µg/ml anti-CD28 (BD Pharmingen) and 1 µg/ml anti-CD49d (BD Pharmingen). In order to capture the CD4^+^ T_CM_ cell phenotype prior to *in vitro* Ag stimulation, anti-CCR7-PE (0.15 µg/ml; R&D Systems) was also included in the culture medium [15]. For each sample/stimulant combination 2×10^5^ cells were added to each of eight wells of the culture plate. GolgiPlug (BD Pharmingen), at a final concentration 1 µg/ml per well, was added for the last 16 h of culture. A negative (anti-human CD28 and CD49d) and a positive [staphylococcal enterotoxin B, 1 µg/ml; (Sigma-Aldrich)] control were included in each assay. After incubation, the cells for each stimulant group were harvested, pooled and transferred to a FACS tube, washed in PBS plus 1% BSA and stained with anti-CD3-PerCP (BD Pharmingen), anti-CD4-PacificBlue (BD Pharmingen), anti-CD8-PacificOrange (Caltag), anti-CD45RO-Alexa700 (Biolegend) and UV Viability dye (Invitrogen). The cells were washed, fixed, and permeabilized with the Cytofix/Cytoperm kit (BD Pharmingen), and stained with anti-IFN-γ-FITC (BD Pharmingen), anti-IL2-Allophycocyanin (BD Pharmingen), and anti-TNF-α-PECy7 (BD Pharmingen). Cells were analyzed using 9-color panels on an LSRII (Becton-Dickinson). Data were analyzed using the FlowJo (Tree Star) software. The frequency of cytokine-producing CD4^+^ T cell subsets in the medium control cultures were subtracted from the corresponding values for the CD4^+^ T cell subsets in the cultures containing Ag.

### Ab ELISA

Ab to CSP were determined by evaluating IgG responses to the Pf CSP R region as measured using standard ELISA with R32LR as the capture Ag and reported in µg/ml [16], [17], [18].

### Statistical Analyses

Prior to statistical analysis any values from the intracellular cytokine staining assay that were less than or equal to zero were adjusted to 0.001. Statistical analyses were performed using GraphPad Prism 4.03 for Windows (GraphPad Software). Groups were compared using the two-tailed Mann Whitney U test. For the data presented in Figure 7, variables were log transformed due to the non-normality of the untransformed data as well as the range (over 3000 fold) of the untransformed data. Spearman's rank correlation co-efficient (SAS version 9.12) was used to assess the agreement between the log of IgG level and log of the percentage of T cells expressing IL-2.

## Results

### PBMC from RTS,S/AS-immunized subjects contain readily detectable populations of CD4^+^ T_E/EM_ and CD4^+^ T_CM_ cells

**Figure 1:**
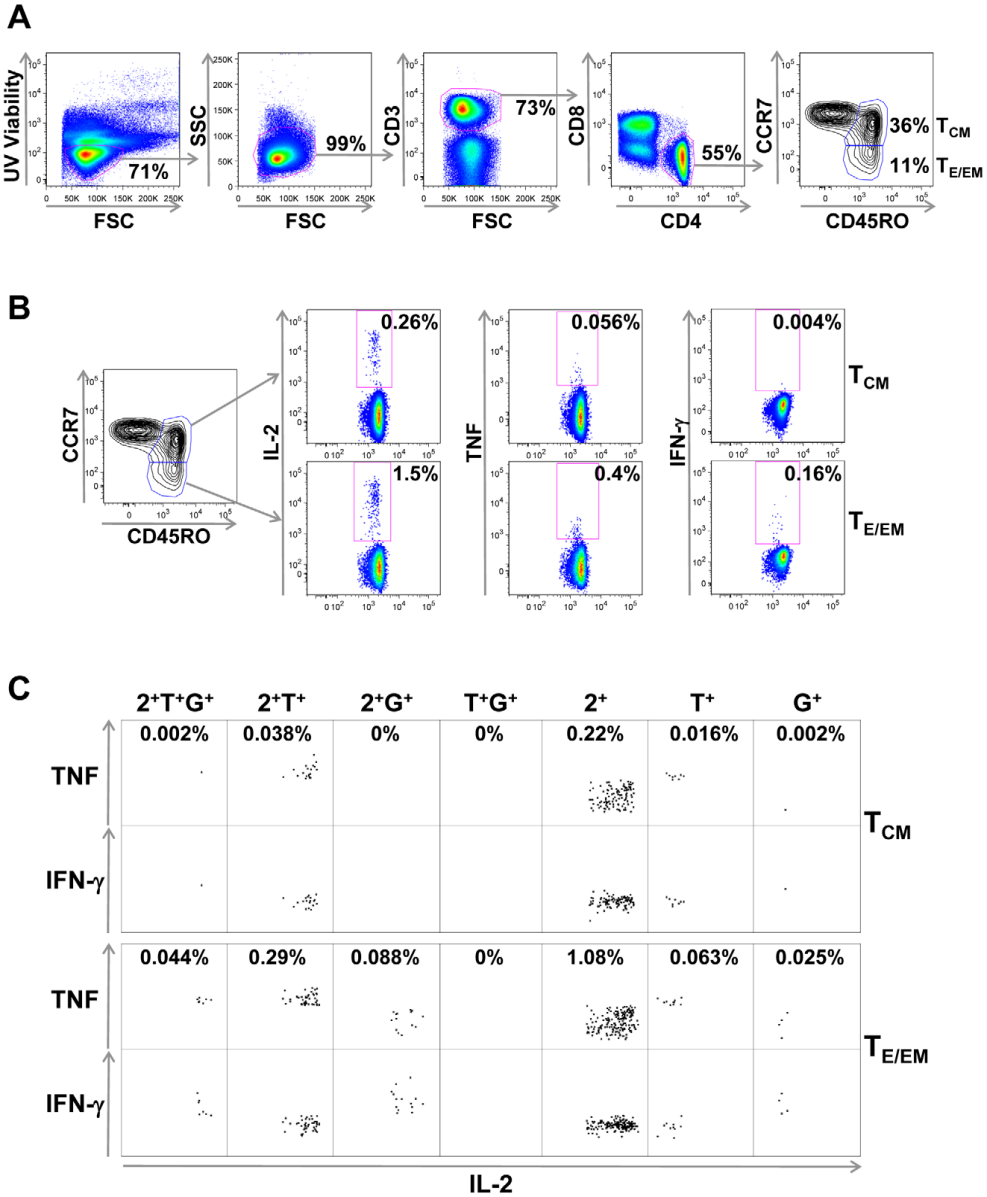
Identification of RTS,S vaccine-induced CSP-specific CD4^+^ T cell memory subsets. PBMCs from an RTS,S-immunized volunteer were stimulated in vitro for 18 h with anti-CD28 and anti-CD49d plus a pool of 2 long contiguous CSP peptides in the presence of PE-conjugated anti-CCR7 Ab. Cells were harvested and surface-labeled for CD3, CD4, CD8, CD45RO and UV viability dye and then stained intra-cellularly for IL-2, TNF-α and IFN-γ. Cells were acquired on an LSR-II flow cytometer and analyzed using FlowJo. (A) Gating strategy used to identify CD4^+^CD45RO^+^CCR7^+^ T_CM_ and CD4^+^CD45RO^+^CCR7^−^ T_E/EM_ cells. Numbers indicate the percentage of cells in each gate. (B) Detection of IL-2, TNF-α and IFN-γ production by CD4^+^ T_CM_ and CD4^+^T_E/EM_ cells. Numbers indicate the percentage of cytokine^+^ cells for each memory cell type. (C) Boolean gating analysis was used to divide cytokine-producing cells into seven distinct populations based on their production of IL-2 (2), TNF-α (T) and IFN-γ (G) in any combination. Each population is shown as two dot plots: the upper is TNF-α vs IL-2 and the lower is IFN-γ vs. IL-2. Numbers indicate the percentage of cytokine^+^ cells for each memory subtype.

PBMC collected at baseline and at each time point after vaccination and challenge were stimulated for 18 h with 2 contiguous 34 and 44-mer CSP peptides and CD4^+^ T cells were then analyzed for the expression of phenotypic surface markers indicative of memory subsets. CD45RO^+^ CD4^+^ T cells were further subdivided into CCR7^+^ T_CM_ and CCR7^−^ T_E/EM_ subsets (Fig. 1A). Because it is conceivable that stimulation with antigen *in vitro* may have induced some CCR7^+^ T_CM_ cells to differentiate into CCR7^−^ T_E/EM_ cells, and Brefeldin A has been shown to interfere with CCR7 expression on activated CD4^+^ T cells [15], we included the fluorescently-labeled CCR7-specific antibodies in the culture medium to capture the CD4^+^ T cell phenotype prior to antigen stimulation. In addition, the short culture period most likely minimized the possibility of *in vitro* differentiation from a CCR7^+^ to a CCR7^−^ T cell subset. The frequencies of CD4^+^ T_CM_ (mean 32%; range 11–58%) and T_E/EM_ cells (mean 9%; range 2–35%) cells were constant for each specific individual over the course of the study. Once we identified the CD4^+^ T_E/EM_ and T_CM_ cell subsets, we then characterized their cytokine production to determine the relative contribution of each subset to protective immunity.

### Protected volunteers generate higher frequencies of IL-2-producing CD4^+^ T_E/EM_ and CD4^+^ T_CM_ cells than non-protected volunteers

**Figure 2:**
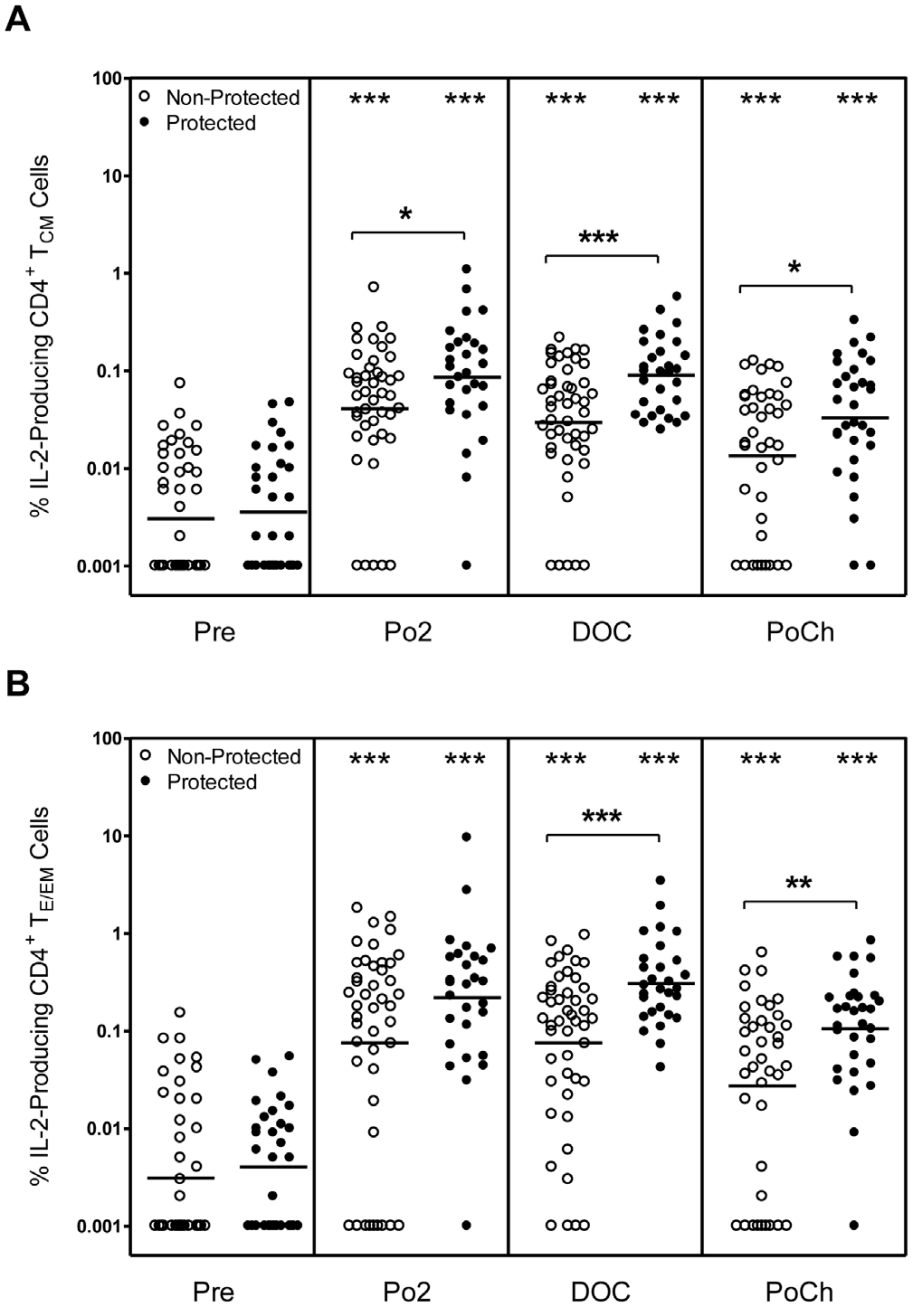
Frequency of CD4^**+**^ T_CM_ and CD4^**+**^ T_E/EM_ cells producing IL-2 upon recall with CSP peptides. PBMC were obtained at the Pre, Po2, DOC and PoCh timepoints from RTS,S-immunized subjects and were incubated for 18 h with co-stimulants (medium control) or co-stimulants plus a pool of 2 long contiguous CSP peptides as described in Methods. Cultures also contained PE-conjugated-CCR7-specific Ab to detect CCR7^+^ cells. Cells were harvested and surface-labeled for CD3, CD4, CD8 and CD45RO and then stained for intracellular detection of IL-2, TNF-α and IFN-γ (see Methods and Fig. 1). Cells were analyzed on an LSR-II flow cytometer to identify IL-2-producing CCR7^+^CD45RO^+^CD4^+^ T_CM_ cells and CCR7^−^CD45RO^+^CD4^+^ T_E/EM_ cells. The frequency of IL-2-producing CD4^+^ T cell subsets in the medium control cultures were subtracted from the corresponding values for the CD4^+^ T cell subsets in the cultures containing Ag. The resulting adjusted values are plotted as [A] the % IL-2 producing CD4^+^ T_CM_ cells and [B] the % IL-2 producing CD4^+^ T_E/EM_ cells for protected (closed symbol) and non-protected (open symbol) subjects. Statistically significant differences between pre-immune and post-vaccination time points are indicated at the top of the graph. Statistically significant differences between values for protected and non-protected subjects are as indicated within the body of the graph. *p<0.05; **p<0.01; ***p<0.001.

**Figure 3:**
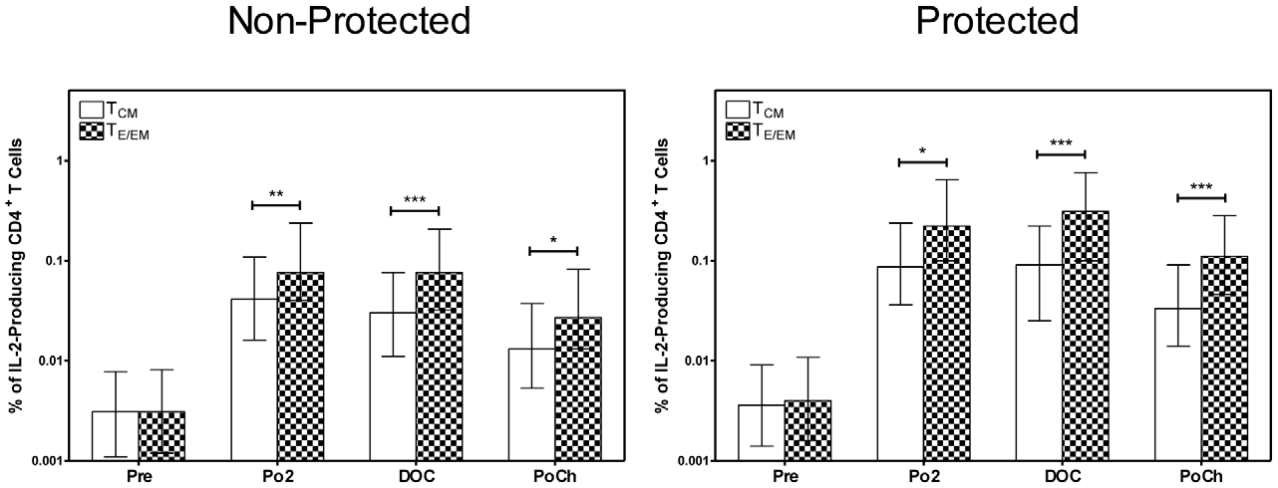
Frequency of CD4^**+**^ T_CM_ and CD4^+^T_E/EM_ cells producing IL-2 upon recall with CSP peptides. PBMC obtained at the indicated time points from RTS,S-immunized subjects were incubated as described for Fig. 2 with CSP peptides as described in Methods. Cells were then analyzed as described in the legend to Fig. 1. The values are plotted as the frequency of IL-2-producing CD4^+^ T_CM_ cells (open bars) and IL-2-producing CD4^+^ T_E/EM_ cells (closed bars) for protected (right-hand panels) and non-protected (left-hand panels) subjects. Error bars indicate 95% confidence intervals. *p<0.05; **p<0.01; ***p<0.001.

In our previous study [8] we showed that the frequency of CD40L^+^CD4^+^ T cells producing IL-2 was higher than those producing TNF-α and IFN-γ. Therefore, to confirm and extend these results we began our study by examining the capacity of CD4^+^ T_E/EM_ and T_CM_ cells to produce IL-2. A typical intracellular staining to detect IL-2, as well as TNF-α and IFN-γ in the CD4^+^ T cell subsets is shown in Figure 1B; Figure 1C shows the Boolean gating analysis to divide cytokine-producing cells into seven distinct populations based on their production of IL-2, TNF-α and IFN-γ in any combination. The cumulative results for IL-2-producing CD4^+^ T_CM_ and T_E/EM_ cells from all of the subjects are shown in Figure 2. The frequency of CSP-specific, IL-2-producing CD4^+^ T_CM_ and T_E/EM_ cells from all time points after vaccination and challenge was significantly higher than the corresponding frequency of cells observed at pre-immunity. Notably, the frequencies of the IL-2 producing CD4^+^ T_E/EM_ and T_CM_ cells from protected subjects were also significantly higher than the corresponding frequencies of cells from the non-protected subjects at most of the time points examined, and the difference was most apparent on the DOC. A study by Sallusto and colleagues [12] demonstrated that CD4^+^ T_CM_ cells responding to polyclonal stimulation produce moderately greater amounts of IL-2 than CD4^+^ T_E/EM_ cells. By contrast, in the present investigation, we show that the percentage of T_E/EM_ cells producing IL-2 in response to CSP peptide stimulation was 2 to 6 fold greater than the percentage of T_CM_ cells (Fig. 3). Differences were statistically significant for both protected and non-protected individuals at all time points. However, as noted in Figure 1, T_CM_ were more abundant than T_E/EM_.

Having determined that the frequencies of CS protein-specific IL-2^+^ CD4^+^ T_CM_ and IL-2^+^ CD4^+^ T_E/EM_ cells were higher for protected compared to non-protected subjects, we asked whether the total numbers of these cells would also be higher in the protected group. Using the total blood lymphocyte counts obtained on the DOC and the frequencies of each cell subset determined by flow cytometry after *in vitro* stimulation with the 2 CSP peptides, we were able to calculate the total number of cells for each subset per microliter of blood. As shown in Table 1, the total number of CD4^+^ T_CM_ cells and of CD4^+^ T_E/EM_ cells capable of producing IL-2 in response to CSP peptides were significantly higher (p<0.003 CD4^+^ T_CM_; p<0.015 CD4^+^ T_E/EM_) in protected as compared to non-protected subjects. Hence, not only the frequencies, but also the total numbers of CSP-specific IL-2^+^ CD4^+^ T_CM_ and IL-2^+^ CD4^+^ T_E/EM_ cells correlated with protection.

**Table 1:** Number of cells per subset on the DOC.

Cell Subset^a^	Protected^b^ (n = 29)	Non-protected^b^ (n = 47)	*P* value^c^
White cells	6083±307	5536±293	0.051
Lymphocytes	1754±84	1725±75	0.68
CD3^+^	1412±79	1390±57	0.78
CD4^+^	912±61	849±39	0.49
CD4^+^ T_CM_	279±28	298±16	0.27
IL-2^+^	0.297±0.042	0.173±0.026	**0.003**
TNF^+^	0.068±0.019	0.053±0.012	0.33
IFN-γ^+^	0.026±0.007	0.028±0.006	0.79
Double Cytokine ( IL-2^+^TNF^+^) Producing T_CM_	0.058±0.015	0.036±0.009	0.16
CD4^+^ T_E/EM_	0.75±0.077	0.93±0.077	0.10
IL-2^+^	0.326±0.063	0.166±0.025	**0.015**
TNF^+^	0.085±0.022	0.047±0.010	**0.062**
IFN-γ^+^	0.058±0.018	0.051±0.023	0.33
Double Cytokine ( IL-2^+^TNF^+^) Producing T_EM_	0.064±0.018	0.032±0.005	0.13

a per µl of peripheral blood.

b mean ± SEM.

c Mann-Whitney *U*-test.

Finally, we asked whether the percentage of subjects generating an IL-2 response significantly above the corresponding pre-immune level was comparable for protected and non-protected subjects. For the purpose of this analysis, a positive post-immune response was defined as being at least 2-fold higher than the corresponding pre-immune value, and at least 2 SD above the group mean of the values for the pre-immune samples. The results showed that the percentage of subjects that manifested a response on the DOC was significantly higher for protected (P) as compared to non-protected (NP) subjects for both CD4^+^ T_E/EM_ cells (97% (P) vs. 62% (NP); p = 0.0007) and CD4^+^ T_CM_ cells (86% (P) vs. 47% (NP); p = 0.0006). These differences were not significant for the post-second immunization cells but there was a strong trend (p = 0.052) for the 2 month post-challenge CD4^+^ T_E/EM_ cells. There was, however, no significant difference between the mean frequencies of DOC IL-2^+^ CD4^+^ T cells from the responding protected and non-protected subjects.

### On the DOC protected subjects have a higher frequency of TNF-α^+^ CD4^+^ T_E/EM_ cells than non-protected subjects

**Figure 4:**
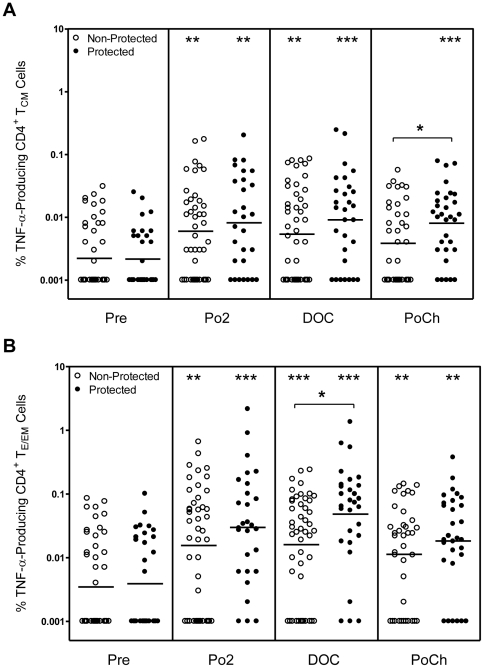
Frequency of CD4^+^T_CM_ and T_E/EM_ cells producing TNF-α upon recall with CSP peptides. PBMC were obtained at the indicated time points from RTS,S-immunized subjects and were incubated as described in Fig. 3 with CSP peptides as described in Methods. Cells were then analyzed as described in the legend to Fig. 1. The values are plotted as [A] the % TNF-α-producing CD4^+^ T_CM_ cells and [B] the % TNF-α-producing CD4^+^ T_E/EM_ cells for protected (closed symbol) and non-protected (open symbol) subjects. Statistically significant differences between pre-immune and post-vaccination time points are indicated at the top of the graph. Statistically significant differences between values for protected and non-protected subjects are as indicated within the body of the graph. *p<0.05; **p<0.01; ***p<0.001.

IL-2 is generally thought to be a growth promoting cytokine whereas effector cytokines such as TNF-α and IFN-γ are thought to mediate parasite killing. Therefore, we also investigated the relative frequencies of TNF-α producing CD4^+^ T_E/EM_ and T_CM_ subsets and the extent to which these frequencies correlate with protection. Both the CD4^+^ T_CM_ and the T_E/EM_ cells from protected and non-protected subjects developed a TNF-α response upon stimulation with CSP peptides, and on the DOC, the protected subjects had a higher frequency of the TNF-α^++^ CD^+^T_E/EM_ cells than the non-protected subjects (Fig. 4). The frequency of the CD4^+^ T_E/EM_ cells producing TNF-α was much greater than that of the CD4^+^ T_CM_ cells (Fig. 4) and these differences were most pronounced for the protected subjects on the DOC. Moreover, as shown in Table 1, there was also a strong trend (p<0.06) toward a higher total number of CSP-specific TNF-α^+^ CD4^+^ T_E/EM_ cells in the protected as compared to the non-protected subjects. Finally, as was observed for the IL-2^+^ CD4^+^ T cells, the percentage of subjects elaborating a positive DOC TNF-α^+^ CD4^+^ T_E/EM_ cell response was significantly higher (p = 0.017) for the protected as compared to the non-protected subjects.

### Only CD4^+^ T_E/EM_ cells make measurable IFN-γ responses

**Figure 5:**
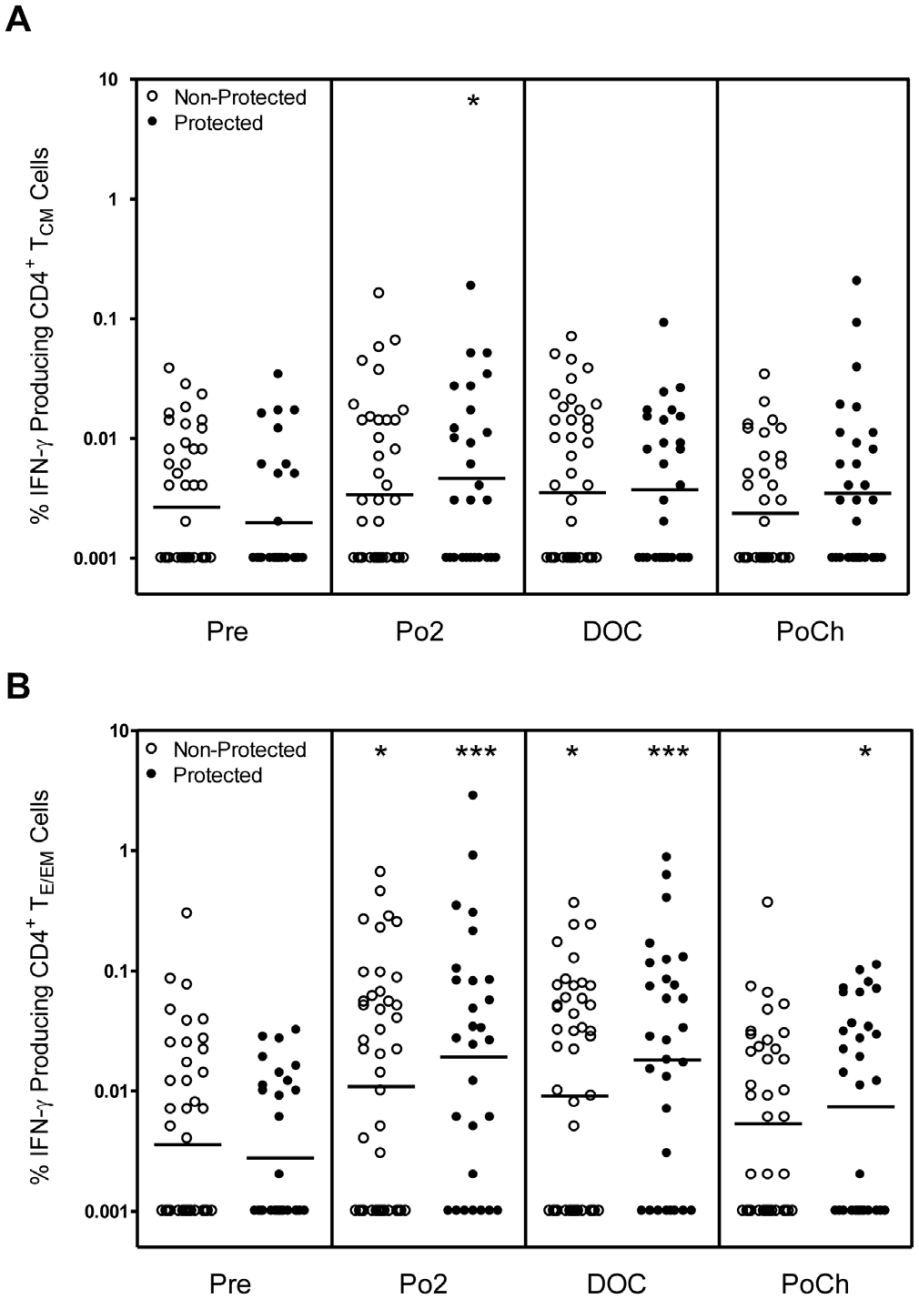
Frequency of CD4^**+**^ T_CM_ and CD4^**+**^T_EM_ cells producing IFN-γ upon recall with CSP peptides. PBMC were obtained at the indicated time points from RTS,S-immunized subjects and were incubated as in Fig. 3 with CSP peptides as described in Methods. Cells were then analyzed as described in the legend to Fig. 1. The resulting values are plotted as the % of [A] IFN-γ producing CD4^+^ T_CM_ cells and [B] IFN-γ producing CD4^+^ T_E/EM_ cells for protected (closed symbol) and non-protected (open symbol) subjects. Statistically significant differences between pre-immune and post-vaccination time points are indicated at the top of the graph. Statistically significant differences between values for protected and non-protected subjects are as indicated within the body of the graph. *p<0.05; **p<0.01; ***p<0.001.

We also measured the frequency of IFN-γ-producing memory CD4^+^ T cell subsets and found that only IFN-γ^+^ T_E/EM_ cells had a post-vaccination frequency, which exceeded that observed at baseline (Fig. 5). Although the mean frequencies of CD4^+^ T_E/EM_ cells producing IFN-γ were relatively low, 15% of volunteers exhibited frequencies of IFN-γ^+^ T_E/EM_ cells ranging between 0.1% to 2.8% of the total CD4^+^ T_E/EM_ population, for both post 2^nd^ and post 3^rd^ vaccination time points. Under no conditions, however, did we observe a significant difference between the mean frequencies of IFN-γ^+^ CD4^+^ T_E/EM_ cells from protected vs. non-protected volunteers.

### Protected subjects have a higher frequency of IL-2^+^ TNF-α^+^ CD4^+^ T cells than non-protected subjects

**Figure 6:**
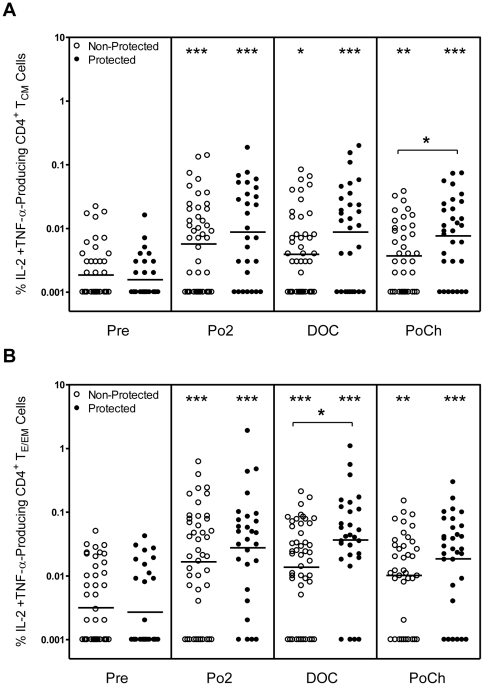
Frequency of CD4^+^ T_CM_ and T_E/EM_ cells producing both IL-2 and TNF-α upon recall with CSP peptides. PBMC were obtained at the indicated time points from RTS,S-immunized subjects and were incubated as described in Fig. 3 with CSP peptides as described in Methods. Cells were then analyzed as described in the legend to Fig. 1. The values are plotted as the % [A] CD4^+^ T_CM_ cells producing both IL-2 and TNF-α and [B] CD4^+^ T_E/EM_ cells producing both IL-2 and TNF-α for protected (closed symbol) and non-protected (open symbol) subjects. Statistically significant differences between pre-immune and post-vaccination time points are indicated at the top of the graph. Statistically significant differences between values for protected and non-protected subjects are as indicated within the body of the graph. *p<0.05; **p<0.01; ***p<0.001.

There is some evidence that poly-functional T cells, i.e., cells producing IL-2 plus one or more effector cytokines, are more efficient at eliminating cellular pathogens than cells producing only one cytokine [19], [20]. Similarly, we previously demonstrated [8] that the frequency of RTS,S-induced CSP peptide-reactive CD4^+^ T cells producing any two immune markers amongst CD40L, IL-2, TNF-α or IFN-γ, was significantly higher on the DOC in protected subjects as compared to non-protected subjects. Therefore, we measured the relative frequencies of memory CD4^+^ T cell subsets capable of producing multiple cytokines. The frequencies of IL-2^+^ IFN-γ^+^ or TNF-α^+^ IFN-γ^+^ CD4^+^ T cells from protected subjects did not differ significantly from those of non-protected subjects (data not shown). As shown in Figure 6, the frequencies of IL-2^+^ TNF-α^+^ T_E/EM_ and T_CM_ cells from both protected and non-protected subjects were, at all time points, significantly higher than the corresponding pre-immune backgrounds. In addition the frequency of IL-2^+^ TNF-α^+^ CD4^+^ T_E/EM_ cells observed on the DOC and of IL-2^+^ TNF-α^+^ CD4^+^ T_CM_ cells observed PoCh were significantly higher in protected vs. non-protected subjects.

### The frequency of CSP-specific, IL-2 producing CD4^+^ T fells correlates with CSP R region-specific Ab titers

**Figure 7:**
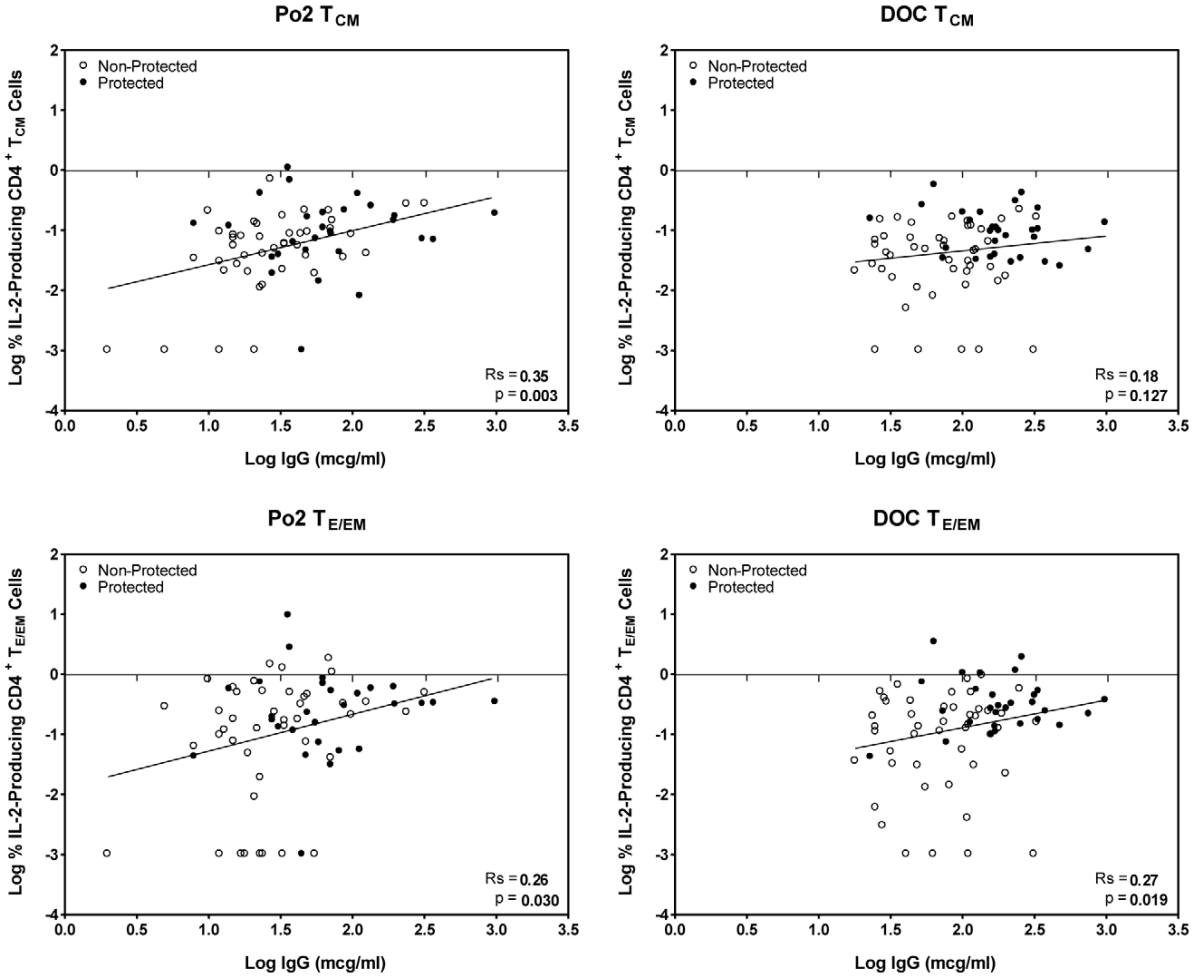
CSP-specific IL-2^+^ T_E/EM_ and T_CM_ CD4^+^ T cells correlate with anti-CSP R region Ab titers. The figures display the log base 10 of the IgG level in micrograms per milliliter versus log of the percent of T cells expressing IL-2 as assayed by flow cytometry. Individuals who were protected from malaria by vaccination are noted with filled circles, while those not protected by open circles. The figure also shows the Spearman's correlation between the log IgG and log percent cells for the combined protected and non-protected individuals as well as a trend line.

It is conceivable that the IL-2 producing CD4^+^ T cells could provide help to promote the differentiation of CSP R region-specific B cells into Ab-producing plasma cells. For example, Litjens et al [21] recently demonstrated a correlation between HBsAg-specific IL-2-producing memory T cells and the generation of IgG-secreting plasma cells. To investigate if the level of CSP R region-specific B cell responses were associated with the magnitude of RTS,S-induced IL-2^+^ CD4^+^ T cells, we performed statistical calculations using Spearman's regression analysis that integrates the levels of CSP R region-specific serum Ab of each volunteer with the corresponding frequency of IL-2 producing CD4^+^ T_E/EM_ and T_CM_ cells. The results (Fig. 7) show statistically significant correlations between the frequency of IL-2 producing CD4^+^ T cells and Ab responses for T_E/EM_ Po2 (Rs = 0.26; p = 0.03) and DOC (Rs = 0.27; p = 0.02) and for T_CM_ Po2 (Rs = 0.35; p = 0.003). There was also, albeit weaker, correlation between the frequency of IL-2^+^TNF-α^+^ CD4 T cells and Ab production (data not shown) but no correlation was observed between Ab levels and total TNF-α^+^ CD4^+^ T cells.

## Discussion

Ag-specific immunologic memory is one of the cardinal features of the immune response and the maintenance of long-lived memory T and B cells is inextricably linked to protracted protective immunity because these memory cells are able to generate a prompt and robust response upon re-encounter with Ag. It has been amply demonstrated in several vaccine systems that long-term protection is often maintained by subsets of memory T cells including T_E/EM_ cells that rapidly produce cytokine upon re-exposure to Ag and T_CM_ cells that have the additional property of being able to undergo homeostatic proliferation in order to maintain the memory pool [1]. The results from our present study demonstrate that RTS,S-induced PBMC contain both CSP-specific CD4^+^ T_E/EM_ and CD4^+^ T_CM_ cells and that both subsets can be re-stimulated *in vitro* with CSP peptides to generate strong IL-2 responses. Notably, not only the frequencies, but also the total number of IL-2-producing CD4^+^ T_E/EM_ and CD4^+^T_CM_ cells, were higher for protected as compared to non-protected volunteers. This result is in agreement with our previous findings [8] that CSP peptide-stimulated PBMC from protected individuals generated a higher frequency of IL-2^+^ Elispots than PBMC from non-protected individuals as well as a higher frequency of CSP-specific CD4^+^ T cells expressing two or more of the CD40L, IL-2, TNF-α and IFN-γ biomarkers. The percentage of subjects generating a positive DOC IL-2^+^ CD4^+^ T cell response was also significantly higher for protected versus non-protected subjects. The lack of responsiveness in some subjects seems to account, in part, for the inability of the RTS,S vaccine to confer protection to 100% of the vaccinees. Whether these differences are due to genetic or other factors will require further investigation.

With regard to effector cytokines, stimulation of CD4^+^ T_E/EM_ cells from protected subjects with CSP peptides induced TNF-α secretion and once again both the frequency (highly significant) and the number (strong trend) of these TNF-α-producing CD4^+^ T_E/EM_ cells were higher for protected vs. non-protected volunteers. By contrast, with the exception of the PoCh time point, the responses of TNF-α-producing CD4^+^ T_CM_ cells did not correlate with protection. Similarly, CD4^+^ T_E/EM_ but not T_CM_ cells developed a relatively weak IFN-γ response, but in no case was there a correlation with protection.

The observation that the frequency of CD4^+^ T_E/EM_ cells producing IL-2 was higher than the frequency of the corresponding population of CD4^+^ T_CM_ cells differs from that of Sallusto et al [12] who showed that polyclonally activated human CD4^+^ T_CM_ produce higher amounts of IL-2 than CD4^+^ T _E/EM_ cells. Other more recent studies [20] have reported that under certain conditions, for example, vaccinia stimulation of human virus-specific CD4^+^ T cells, IL-2 is produced predominantly by CD4^+^ T_E/EM_ cells. Moreover, Divekar et al [22] demonstrated that humans immunized with protein vaccines such as tetanus and diphtheria, also have a higher frequency of IL-2-producing CD4^+^ T_E/EM_ cells than IFN-γ-secreting T_E/EM_ cells. Seder et al [5] have proposed a linear differentiation model for CD4^+^ T cells in which naïve T cells proceed along a pathway that begins with CD4^+^ T_CM_ cells producing only IL-2 or TNF-α followed by T_CM_ cells that produce both IL-2 and TNF-α and subsequently by cells that begin to also produce IFN-γ. They further state that this model is most evident following vaccination with protein plus adjuvant. It is conceivable, therefore, that CD4^+^ T_E/EM_ cells from persons vaccinated with a protein Ag such as RTS,S produce predominately IL-2 on the DOC because they might be only at the initial stages of the contraction phase. Alternatively, it should be emphasized that our analyses were performed on peripheral blood cells that may include re-circulating T_CM_ cells that have transiently down-regulated CCR7 in order to leave the lymph node and enter the blood stream.

In the present study, IL-2 was the predominant cytokine produced by both memory phenotype CD4^+^ T cells upon Ag recall *in vitro* and both the frequencies and the numbers of the IL-2^+^ CD4^+^ T_CM_ and T_E/EM_ cells also correlated strongly with protection. There are several mechanisms that might explain the latter observation. For example, IL-2 could act as an autocrine or paracrine growth factor to promote the expansion of cells producing effector cytokines such as TNF-α. We showed previously that IL-2-producing CD4^+^ T cells also express CD40L [8] and hence they may have promoted a protective immune response by licensing DCs to produce cytokines and by inducing the differentiation of B cells through CD40L-CD40 interactions [23]. We have also demonstrated recently that CSP R region-specific Abs make a strong and significant contribution to RTS,S-induced protection [8] and IL-2, therefore, might contribute to protection by directly promoting the growth and differentiation of R region-specific B cells [21], [24]. Similarly, IL-2 may act as a growth factor for follicular Th cells that induce B cells to undergo rapid proliferation and hence drive the germinal center reaction [23]. In support of this mechanism, the results in Figure 7 demonstrate a direct correlation between the frequency of IL-2-producing CD4^+^ T cells and the titers of CSP R region-specific antibodies in the same individual. In agreement with this, Galli et al [25] also reported recently that immunization of humans with an avian influenza vaccine in the MF59 adjuvant induces virus-specific CD4^+^ T cells that produce primarily an IL-2^+^ IFN-γ^−^ phenotype. In addition, the frequency of virus-specific total CD4^+^ T cells induced with one dose of vaccine predicted the rise and long-term maintenance of neutralizing antibodies after booster immunization.

The result in the present study differs, however, from that reported previously [8], which found no correlation between RTS,S-induced humoral and cellular responses. Several factors might provide at least a partial explanation for this discrepancy. In the previous study, we used the total frequency of CD4^+^ T cells expressing any two of the CD40L, IL-2, TNF-α and IFN-γ biomarkers in the antibody correlation studies, whereas in the present study we considered only IL-2 responses. The discrepancy could also be due to the use of different culture conditions including cell densities and antigens, (15-mer versus 35-mer and 40-mer peptides), used for *in vitro* re-stimulation. We also analyzed the combined effect of both antibody and frequency of IL-2^+^ CD4^+^ T cells as a potential correlate of protection, but there was no increase in correlation relative to that obtained with antibody alone (data not shown). This was likely due to the already very strong correlation of antibody levels with protection.

The current results also show that both the frequency and the number (strong trend) of CD4^+^ T_E/EM_ cells producing TNF-α were higher on the DOC in protected vs. non-protected subjects. This finding is in agreement with previous reports [26], [27] that TNF-α can suppress the growth of exo-erythrocytic forms of the malaria parasite *in vitro* [28] and *in vivo* [26], [27]. There is also evidence [19] that poly-functional T cells producing more than one cytokine provide greater protection against infection than T cells secreting only one cytokine. In the present study, we also identified a population of CD4^+^ T_E/EM_ cells that were producing both IL-2 and TNF-α and the frequency of these cells on the DOC also correlated with protection. It is conceivable that IL-2 was acting as an autocrine growth factor to drive the expansion of these cells, while TNF-α was mediating their effector function. These results are also very similar to those of Huaman et al [29] who examined cytokine responses by CD4^+^ T cells in humans immunized three times with the malaria antigen AMA-1formulated in Alhydrogel. As in our study, they found that the highest frequency of AMA-1-specific, multifunctional CD4^+^ T cells were IL-2^+^TNF-α^+^ double producers and they further demonstrated that the frequency of IL-2 single producers increased after immunization. Similarly, they also detected both T_CM_ and T_EM_ cells even as early as 7 days after the primary immunization, although they did not characterize cytokine production by these cells. In the present study, it is noteworthy that the correlation of protection with the frequency of CD4^+^ T cells making TNF-α (Fig. 4) shifted from CD4^+^ T_E/EM_ cells at the DOC to CD4^+^ T_CM_ cells at 2 mo PoCh. A similar shift was also observed for CD4^+^ T cells producing both IL-2 and TNF-α (Fig. 6). It is likely that on the DOC there was still an abundance of CSP-specific effector-like cells capable of elaborating TNF-α to protect against the parasite. By contrast, by 2 mo PoCh, CD4^+^ T_CM_ cells would likely exceed the number the effector-like cells and hence be the more critical population capable of differentiating into mediators of protection. A similar conclusion regarding the key role of central memory T cells was reported previously by Keating et al [30] who studied cultured Elispot cytokine responses using PBMC from donors immunized in a prime-boost regimen with the malaria {multi-epitope plus TRAP} antigen.

The results of both this and our previous study [8] demonstrate that IL-2 is the predominant cytokine elaborated by RTS,S-induced CD4 T cells upon *in vitro* recall with CSP peptides. By contrast, only CD4^+^ T_E/EM_ cells from a small percentage of volunteers produced IFN-γ in response to CSP peptides. This is in agreement with other reports [12], [13], [14] that CD4^+^ T_E/EM_ cells are the main producers of effector cytokines such as IFN-γ. The present IFN-γ results differ, however, from our previously published studies [8], [31]. In the most recently published study we demonstrated that the frequency of CD4^+^ T cells expressing IFN-γ plus another biomarker (eg. CD40L) was higher in protected than in non-protected subjects [8]. The reasons for this discrepancy is unclear, but may be due to the use of different biomarkers (CCR7 vs. CD40L) and different culture conditions, as described above (vide ante). In the present study we added CCR7-specific Ab directly to the cell cultures. Other investigators have reported [15] adding CCR7-specific Ab to cultures and demonstrating that the proportion of CCR7^+^ and CCR7^−^ CD4^+^ T cells producing IL-2 and IFN-γ following SEB stimulation was similar to those detected in subsets purified by cell sorting. In addition, we also measured post re-challenge IFN-γ production by total CD4^+^ T cells from one subject in the presence and absence of anti-CCR7 and found that the magnitude of the IFN-γ response in the presence of anti-CCR7 was 81% of the analogous responses in the absence of anti-CCR7. Consequently, the presence of anti-CCR7 did not appear to markedly interfere with the IFN-γ response of the cultured CD4^+^ T cells. In the study by Sun et al [31], apart from different culture conditions, the RTS,S was administered according to a different regimen of vaccination, 0, 1 and 7 months, which might have generated stronger CSP peptide-specific IFN-γ^+^CD4^+^ and CD8^+^ T cell responses.

Thirty two subjects from the present RTS,S vaccine trial were protected against the primary challenge; 18 subjects returned for a re-challenge 5 months after the initial challenge, and 8/18 were protected against the secondary challenge. At the day of re-challenge, the frequency of CD4^+^ T cells capable of generating an IL-2 recall response no longer correlated with protection. This result is in agreement with the previous finding [8] that on the day of re-challenge only CS protein-specific CD4^+^ T cells expressing IFN-γ plus one other biomarker had higher levels in protected than in the 5 out of 10 non-protected subjects analyzed. The lack of correlation for IL-2 may have been due to the low number of subjects participating in the re-challenge. In addition, at this much later time point, alternative populations of cells and / or mechanisms may have been involved in mediating protection.

In summary, PBMC from RTS,S/AS vaccinated subjects contained both CD4^+^ T_CM_ and CD4^+^ T_E/EM_ cells that could be re-called *in vitro* with CSP peptides to generate strong IL-2 responses. Both the frequency and the total number of memory CD4^+^ T cells producing IL-2 correlated with protection, indicating that IL-2 may promote B cell and effector T cell responses. In support of this notion, the frequency of IL-2-producing memory CD4^+^ T cells correlated with the CSP-specific Ab titers within corresponding individuals. Immunization with RTS,S/AS also induced a higher frequency of TNF-α producing CD4^+^T_E/EM_ cells in protected as compared to non-protected subjects. Consequently, TNF-α likely also contributes to RTS,S/AS-induced protective immunity.

## References

[1] van Leeuwen EM, Sprent J, Surh CD (2009). Generation and maintenance of memory CD4(+) T Cells.. Curr Opin Immunol.

[2] MacLeod MK, Clambey ET, Kappler JW, Marrack P (2009). CD4 memory T cells: what are they and what can they do?. Semin Immunol.

[3] Romero P, Zippelius A, Kurth I, Pittet MJ, Touvrey C (2007). Four functionally distinct populations of human effector-memory CD8+ T lymphocytes.. J Immunol.

[4] Miller JD, van der Most RG, Akondy RS, Glidewell JT, Albott S (2008). Human effector and memory CD8+ T cell responses to smallpox and yellow fever vaccines.. Immunity.

[5] Seder RA, Darrah PA, Roederer M (2008). T-cell quality in memory and protection: implications for vaccine design.. Nat Rev Immunol.

[6] Doolan DL, Martinez-Alier N (2006). Immune response to pre-erythrocytic stages of malaria parasites.. Curr Mol Med.

[7] Garcon N, Heppner DG, Cohen J (2003). Development of RTS,S/AS02: a purified subunit-based malaria vaccine candidate formulated with a novel adjuvant.. Expert Rev Vaccines.

[8] Kester KE, Cummings JF, Ofori-Anyinam O, Ockenhouse CF, Krzych U (2009). Randomized, double-blind, phase 2a trial of falciparum malaria vaccines RTS,S/AS01B and RTS,S/AS02A in malaria-naive adults: safety, efficacy, and immunologic associates of protection.. J Infect Dis.

[9] Alonso PL, Sacarlal J, Aponte JJ, Leach A, Macete E (2004). Efficacy of the RTS,S/AS02A vaccine against Plasmodium falciparum infection and disease in young African children: randomised controlled trial.. Lancet.

[10] Aponte JJ, Aide P, Renom M, Mandomando I, Bassat Q (2007). Safety of the RTS,S/AS02D candidate malaria vaccine in infants living in a highly endemic area of Mozambique: a double blind randomised controlled phase I/IIb trial.. Lancet.

[11] Sacarlal J, Aide P, Aponte JJ, Renom M, Leach A (2009). Long-term safety and efficacy of the RTS,S/AS02A malaria vaccine in Mozambican children.. J Infect Dis.

[12] Sallusto F, Lenig D, Forster R, Lipp M, Lanzavecchia A (1999). Two subsets of memory T lymphocytes with distinct homing potentials and effector functions.. Nature.

[13] Harari A, Vallelian F, Meylan PR, Pantaleo G (2005). Functional heterogeneity of memory CD4 T cell responses in different conditions of antigen exposure and persistence.. J Immunol.

[14] Sun Y, Schmitz JE, Acierno PM, Santra S, Subbramanian RA (2005). Dysfunction of simian immunodeficiency virus/simian human immunodeficiency virus-induced IL-2 expression by central memory CD4+ T lymphocytes.. J Immunol.

[15] Stubbe M, Vanderheyde N, Goldman M, Marchant A (2006). Antigen-specific central memory CD4+ T lymphocytes produce multiple cytokines and proliferate in vivo in humans.. J Immunol.

[16] Folena-Wasserman G, Inacker R, Rosenbloom J (1987). Assay, purification and characterization of a recombinant malaria circumsporozoite fusion protein by high-performance liquid chromatography.. J Chromatogr.

[17] Ballou WR, Hoffman SL, Sherwood JA, Hollingdale MR, Neva FA (1987). Safety and efficacy of a recombinant DNA Plasmodium falciparum sporozoite vaccine.. Lancet.

[18] Wirtz RA, Ballou WR, Schneider I, Chedid L, Gross MJ (1987). Plasmodium falciparum: immunogenicity of circumsporozoite protein constructs produced in Escherichia coli.. Exp Parasitol.

[19] Darrah PA, Patel DT, De Luca PM, Lindsay RW, Davey DF (2007). Multifunctional TH1 cells define a correlate of vaccine-mediated protection against Leishmania major.. Nat Med.

[20] Kannanganat S, Ibegbu C, Chennareddi L, Robinson HL, Amara RR (2007). Multiple-cytokine-producing antiviral CD4 T cells are functionally superior to single-cytokine-producing cells.. J Virol.

[21] Litjens NH, Huisman M, Hijdra D, Lambrecht BM, Stittelaar KJ (2008). IL-2 producing memory CD4+ T lymphocytes are closely associated with the generation of IgG-secreting plasma cells.. J Immunol.

[22] Divekar AA, Zaiss DM, Lee FE, Liu D, Topham DJ (2006). Protein vaccines induce uncommitted IL-2-secreting human and mouse CD4 T cells, whereas infections induce more IFN-gamma-secreting cells.. J Immunol.

[23] Batista FD, Harwood NE (2009). The who, how and where of antigen presentation to B cells.. Nat Rev Immunol.

[24] Parker DC (1993). T cell-dependent B cell activation.. Annu Rev Immunol.

[25] Galli G, Medini D, Borgogni E, Zedda L, Bardelli M (2009). Adjuvanted H5N1 vaccine induces early CD4+ T cell response that predicts long-term persistence of protective antibody levels.. Proc Natl Acad Sci U S A.

[26] Mazier D, Renia L, Nussler A, Pied S, Marussig M (1990). Hepatic phase of malaria is the target of cellular mechanisms induced by the previous and the subsequent stages. A crucial role for liver nonparenchymal cells.. Immunol Lett.

[27] Korten S, Anderson RJ, Hannan CM, Sheu EG, Sinden R (2005). Invariant Valpha14 chain NKT cells promote Plasmodium berghei circumsporozoite protein-specific gamma interferon- and tumor necrosis factor alpha-producing CD8+ T cells in the liver after poxvirus vaccination of mice.. Infect Immun.

[28] Depinay N, Franetich JF, Gruner AC, Mauduit M, Chavatte JM Inhibitory Effect of TNF-alpha on Malaria Pre-Erythrocytic Stage Development: Influence of Host Hepatocyte/Parasite Combinations.. PLoS One.

[29] Huaman MC, Mullen GE, Long CA, Mahanty S (2009). Plasmodium falciparum apical membrane antigen 1 vaccine elicits multifunctional CD4 cytokine-producing and memory T cells.. Vaccine.

[30] Keating SM, Bejon P, Berthoud T, Vuola JM, Todryk S (2005). Durable human memory T cells quantifiable by cultured enzyme-linked immunospot assays are induced by heterologous prime boost immunization and correlate with protection against malaria.. J Immunol.

[31] Sun P, Schwenk R, White K, Stoute JA, Cohen J (2003). Protective immunity induced with malaria vaccine, RTS,S, is linked to Plasmodium falciparum circumsporozoite protein-specific CD4+ and CD8+ T cells producing IFN-gamma.. J Immunol.

